# Correction to “Alternative RNA Splicing of the *GIT1* Gene Is Associated With Neuroendocrine Prostate Cancer”

**DOI:** 10.1111/cas.70140

**Published:** 2025-07-09

A. R. Lee, Y. Gan, N. Xie, V. R. Ramnarine, J. M. Lovnicki, and X. Dong, “Alternative RNA Splicing of the *GIT1* Gene Is Associated With Neuroendocrine Prostate Cancer,” *Cancer Science* 110, no. 1 (2019): 245–255, https://doi.org/10.1111/cas.13869.

Concerns were raised by a third party regarding image panels with insets without any magnification or scale bar in Figure 2A. The authors acknowledged that these insets were cropped and amplified from the original images; therefore, no scale bar was inserted. The unamplified insets were replaced by the 10× magnified panels accordingly.

The corrected Figure 2A and its corrected figure caption are as follows:

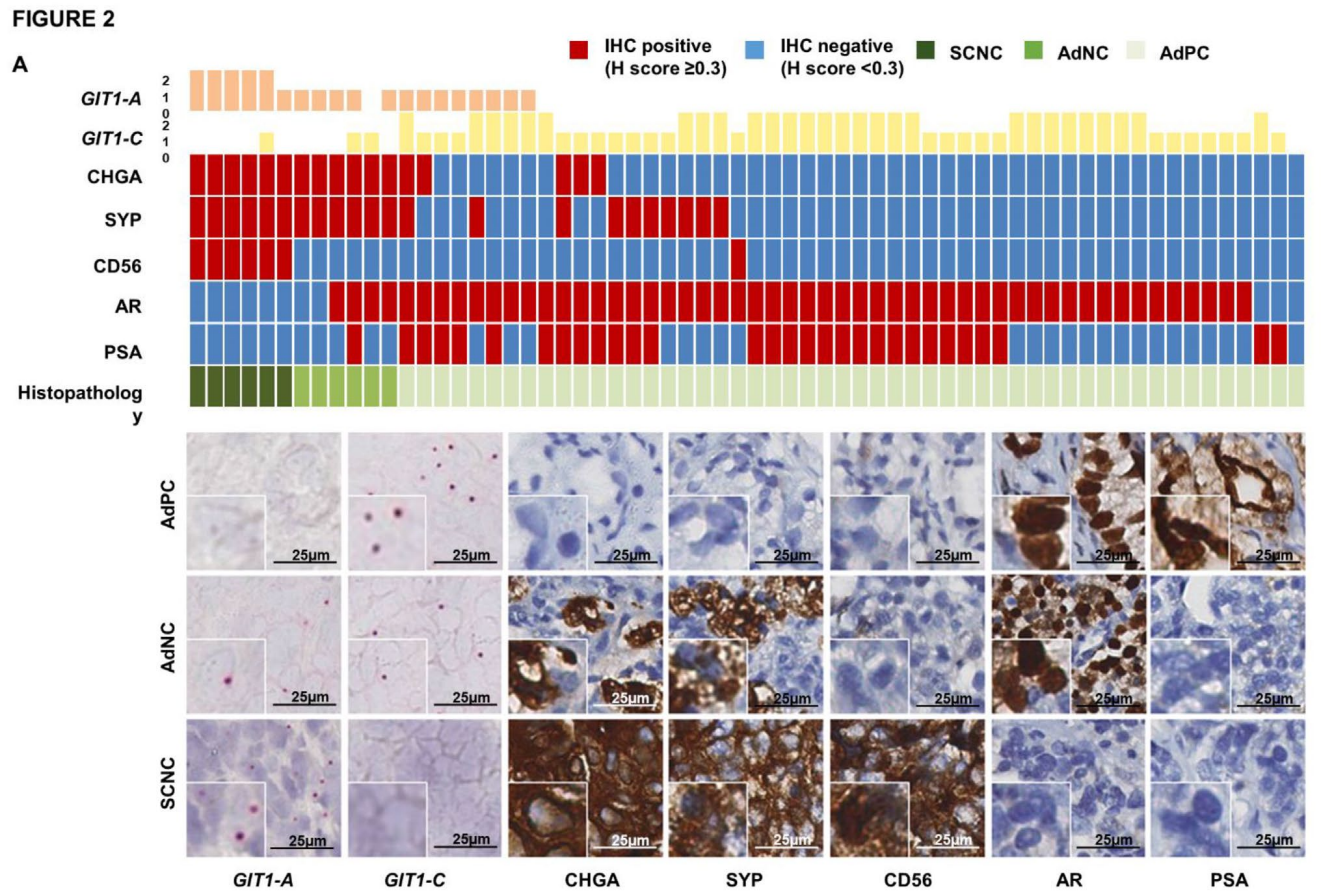

**Figure 2**

RNA splicing of *GIT1* is associated with clinical NEPC tumors. A, RISH probes targeting the exons 7/8 or exons 7/9 junction were created to detect GIT1‐A or GIT1‐C, respectively, in a human CRPC TMA (n = 64 cores). TMA was stained against CHGA, SYP, CD56, AR, and PSA by immunohistochemistry (IHC). Columns in the heatmap represent one of 64 cores. One representative core from each of the histologically diagnosed AdPC (n = 52), AdNC (n = 6), and SCNC (n = 6) cores is shown. Scale bars represent 25 μm. Insets represent cropped and 10× amplified images.

In addition, the figure captions of Figures 3A and 5 have also been revised, as follows:

**Figure 3**

SRRM4 regulates RNA splicing of *GIT1*. A, Matched TMA cores are shown to represent the associations of the expressions of SRRM4 with GIT1‐A and GIT1‐C. Scale bars represent 25 μm. Insets represent cropped and 10× amplified images.

**Figure 5**

Differential functions of the GIT1 splice variants in FA stability. DU145 stable cell lines overexpressing GIT1‐A, GIT1‐C, or empty vector were seeded on coverslips and serum‐starved. They were treated with 10 μmol/L nocodazole for 4 h, subsequently washed away, and replaced with serum‐containing medium. Cells were fixed at 0 or 120 min after the washout, costained against GIT1 and vinculin, and then mounted with DAPI staining mount. Cells were imaged using a Zeiss AxioObserver Z1 (Carl Zeiss AG; Oberkochen, Germany) microscope, where the scale bar represents 10 μm. Insets represent cropped and 10× amplified images. Arrowheads indicate FA complexes. Overlapping signals between GIT1 and vinculin appear yellow. Overlapping of the two signals in a cross section (indicated by white line) of FA complexes was profiled by the ZEN program. All experiments were repeated three times. FA, focal adhesion; GIT1, G‐protein‐coupled receptor kinase‐interacting protein 1; IF, immunofluorescence; ZEN, ZEISS efficient navigation.

